# The role of interferon signaling in neurodegeneration and neuropsychiatric disorders

**DOI:** 10.3389/fpsyt.2024.1480438

**Published:** 2024-10-03

**Authors:** Daniel W. Sirkis, Alexis P. Oddi, Caroline Jonson, Luke W. Bonham, Phuong T. Hoang, Jennifer S. Yokoyama

**Affiliations:** ^1^ Memory and Aging Center, Department of Neurology, Weill Institute for Neurosciences, University of California, San Francisco, San Francisco, CA, United States; ^2^ Center for Alzheimer’s and Related Dementias, National Institutes of Health, Bethesda, MD, United States; ^3^ DataTecnica LLC, Washington, DC, United States; ^4^ Department of Radiology and Biomedical Imaging, University of California, San Francisco, San Francisco, CA, United States; ^5^ Movement Disorders and Neuromodulation Center, Department of Neurology, Weill Institute for Neurosciences, University of California, San Francisco, San Francisco, CA, United States; ^6^ Global Brain Health Institute, University of California, San Francisco, San Francisco, CA, United States

**Keywords:** interferon, neurodegeneration, Alzheimer’s disease, Parkinson’s disease, TDP-43, C9orf72, neuropsychiatric disease, autoimmune disease

## Abstract

Recent advances in transcriptomics research have uncovered heightened interferon (IFN) responses in neurodegenerative diseases including Alzheimer’s disease, primary tauopathy, Parkinson’s disease, TDP-43 proteinopathy, and related mouse models. Augmented IFN signaling is now relatively well established for microglia in these contexts, but emerging work has highlighted a novel role for IFN-responsive T cells in the brain and peripheral blood in some types of neurodegeneration. These findings complement a body of literature implicating dysregulated IFN signaling in neuropsychiatric disorders including major depression and post-traumatic stress disorder. In this review, we will characterize and integrate advances in our understanding of IFN responses in neurodegenerative and neuropsychiatric disease, discuss how sex and ancestry modulate the IFN response, and examine potential mechanistic explanations for the upregulation of antiviral-like IFN signaling pathways in these seemingly non-viral neurological and psychiatric disorders.

## Introduction

**Figure 1:**
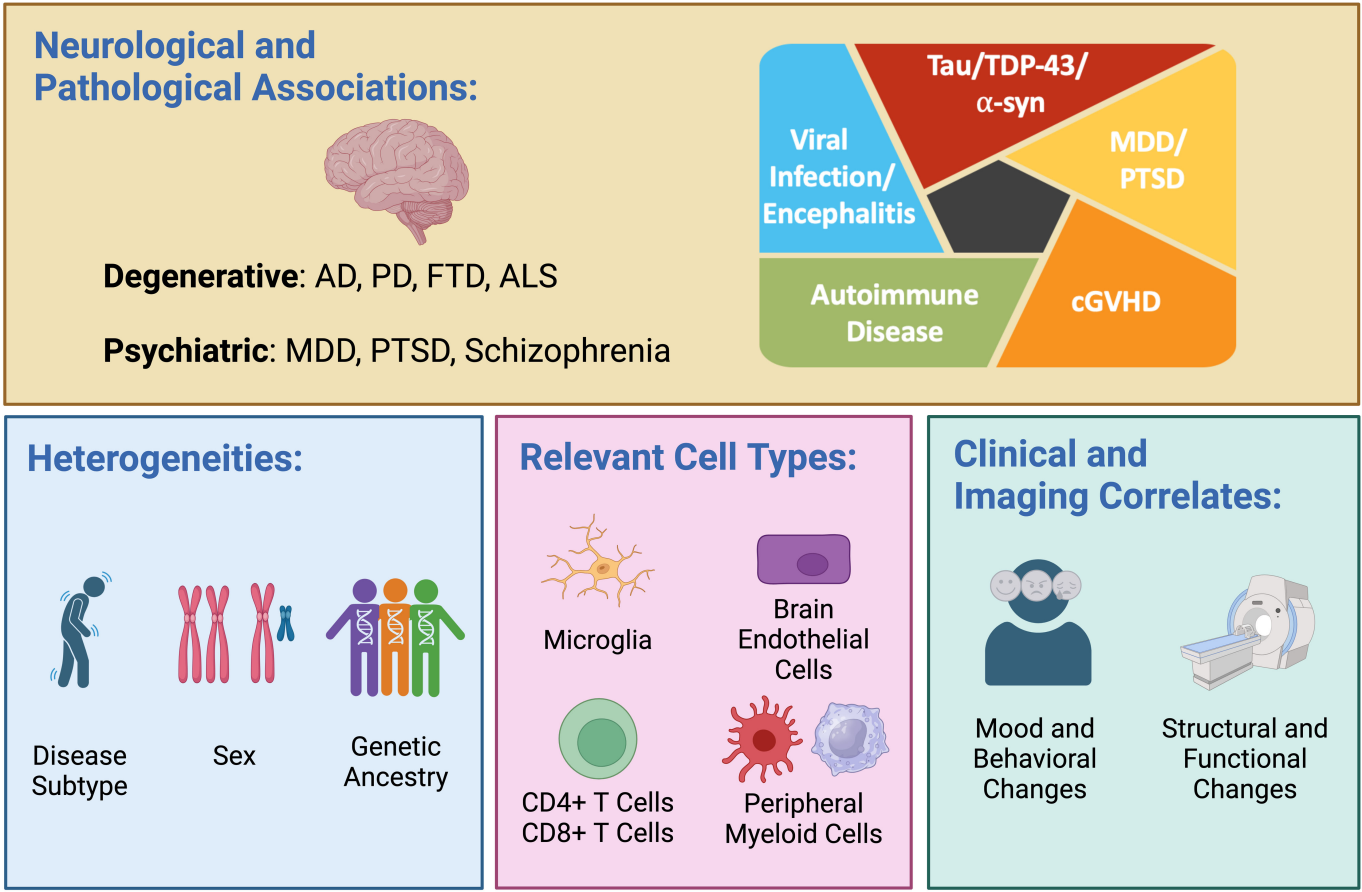
Dysregulated interferon (IFN) responses are observed in a variety of neurodegenerative diseases—including Alzheimer’s disease (AD), Parkinson’s disease (PD), frontotemporal dementia (FTD), and amyotrophic lateral sclerosis (ALS)—and across multiple proteinopathies, including those driven by tau, TDP-43 and α-synuclein (α-syn). Altered IFN signaling is also observed in psychiatric diseases, including major depressive disorder (MDD), post-traumatic stress disorder (PTSD), and schizophrenia (upper panel). A heightened IFN response manifesting as expansion of highly IFN-responsive T cells has been observed in early-onset AD, viral encephalitis, autoimmune disease, and chronic graft versus host disease (cGVHD; upper panel, right). Important biological variables that may modulate the IFN response include disease subtype, sex, and genetic ancestry (lower panel, left). Relevant cell types mediating dysregulated IFN signaling in disease include brain-resident microglia, brain endothelial cells, CD4 and CD8 T cells, and peripheral myeloid cells (lower panel, middle). The use of recombinant type I IFN is associated with cognitive and behavioral changes as well as structural and functional neuroimaging changes (lower panel, right). Created in BioRender. Yokoyama, J. (2024) BioRender.com/n80o218.

The last five years have witnessed impressive growth in the number of publications dissecting the role of interferon (IFN) signaling in neurodegenerative disease. Although the earliest explorations of IFN production in Alzheimer’s disease (AD) occurred more than 40 years ago (1), in this review we will focus primarily on research conducted in the past decade, using the pioneering work of Roy and colleagues (2) as a starting point. We will summarize recent advances in our understanding of the role of dysregulated IFN signaling in AD, primary tauopathy, TAR DNA-binding protein 43 (TDP-43) proteinopathy including cases associated with *C9orf72* hexanucleotide repeat expansion (HRE), and Parkinson’s disease (PD). We will also explore evidence for augmented IFN responses in several neuropsychiatric disorders and discuss whether IFN signaling is likely to play a role in cognitive dysfunction in either neurodegenerative or neuropsychiatric disease. Finally, we will highlight important yet understudied biological modulators of the IFN response, including sex and ancestry. We will conclude by highlighting the most important open questions facing the field, such as which IFN-responsive cell types (e.g., microglia, T cells) are most likely to contribute to pathogenesis and whether targeting IFN signaling represents a promising avenue for modifying pathobiology in neurodegenerative or neuropsychiatric disease (see accompanying **Figure 1**, **Table 1** for a summary of topics discussed and callouts to references of special importance).

**Table 1:** Key studies implicating altered interferon responses in neurodegenerative diseases and neuropsychiatric disorders.

Topic	Key references	Main findings	Study method(s)	Cell type(s) implicated	Proposed direction of interferon (IFN) signaling/response genes
NEURODEGENERATIVE AND NEUROPSYCHIATRIC DISORDERS					
Alzheimer’s disease (AD)	Roy et al., 2020 (2)	Microglia expressing ISGs respond to nucleic acid-containing Aβ fibrils in AD mouse models.	RNA-seq	Microglia	Upregulation of type I IFN response genes in mice with Aβ pathology
	Sirkis & Warly Solsberg et al., 2024 (19)	Expansion of interferon-responsive CD4 T cells in early-onset AD driven by females.	scRNA-seq, ddPCR	CD4 T cells	Upregulation of type I IFN response genes across early-onset AD PBMCs and late-onset AD CSF immune cells
Amyotrophic lateral sclerosis (ALS), frontotemporal dementia (FTD)	McCauley et al., 2020 (30)	Lymphoid and myeloid cells from *C9orf72*-deficient mice show an enhanced type I IFN response signature.	RNA-seq	T cells, myeloid cells	Increased expression of type I IFN genes in lymphoid and myeloid cells (mice); whole blood, myeloid cells, and brain from C9-ALS/FTD
	Bonham, Geier, & Sirkis et al., 2023 (31)	Symptomatic *C9orf72* HRE carriers have global upregulation of TEs in peripheral blood; peripheral *L1HS* expression associates with thalamic nuclei volumes.	RNA-seq; neuroimaging	Peripheral blood cells	Increased type I IFN gene expression and TEs (whole blood and PBMCs) in symptomatic *C9orf72* HRE carriers
	Yu et al., 2020 (46)	Mislocalization of TDP-43 to mitochondria promotes mtDNA release into cytoplasm, triggering cGAS/STING and IFN pathway activation. Deletion of *Sting* in mouse ALS model improves outcomes.	qPCR	Mouse neuronal cell lines; iPSC motor neurons	Increased type I IFN response gene expression in cortex and spinal cord; dependent on *Sting* in TDP-43 mouse models
Parkinson’s disease (PD)	Garretti et al., 2023 (64)	In mouse model expressing DRB1*15:01, immunization against α-syn_32-46_ results in enteric neuron loss potentially mediated by CD4 T cells.	RNA-seq	CD4 T cells; granulocytes	Upregulation of innate and adaptive immune responses, including IFN-stimulated genes in α-syn_32-46_-immunized HLA mice
Major depressive disorder (MDD)	Mostafavi et al., 2014 (70)	Analysis of whole-blood RNA-seq data from MDD patients reveals increased expression of IFN α/β pathway genes, not explained after controlling for confounding diagnoses and/or medications	RNA-seq	Peripheral blood cells	Increased expression of IFN-α and IFN-β signaling genes is associated with MDD
Post-traumatic stress disorder (PTSD)	Breen et al., 2018 (72)	Type I IFN signaling response pathways are altered in PTSD patients.	Network mega-analysis	Peripheral blood cells	Increased expression of type I IFN signaling genes in combat-related traumas; decreased in men with PTSD related to interpersonal trauma
HETEROGENEITIES					
Sex differences	Sayed et al., 2021 (82)	In female tau P301S–TREM2 R47H mice, an IFN-responsive microglia cluster was expanded relative to female mice expressing only the P301S or R47H alleles, or controls.	scRNA-seq	Microglia	Expansion of IFN-responsive microglia in tauopathy mouse model expressing *TREM2* R47H variant
Host genetic variation	Yang et al., 2021 (105)	Background genetic variation in AD mouse models regulates microglial subpopulations.	scRNA-seq	Microglia	IFN-responsive microglia are enriched in APP/PS1 mice, but only on PWK background
CLINICAL & IMAGING CORRELATES					
IFN-α treatment	Nettis et al., 2021 (133)	In healthy participants, IFN-α administration affects T1-relaxation values for hippocampus; T1 values correlated with markers of decreased BBB integrity.	Quantitative MRI	-	-
IFN-β treatment	Coch et al., 2019 (132)	In healthy participants, IFN-β administration decreases functional activity in ventral striatum during reward processing tasks and in amygdala during emotion-recognition tasks.	Functional MRI	-	-

α-syn, α-synuclein; Aβ, amyloid-β; BBB, blood-brain barrier; CSF, cerebrospinal fluid; ddPCR, droplet digital PCR; HLA, human leukocyte antigen; HRE, hexanucleotide repeat expansion; iPSCs, induced pluripotent stem cells; ISGs, interferon-stimulated genes; MRI, magnetic resonance imaging; mtDNA, mitochondrial DNA; PBMCs, peripheral blood mononuclear cells; qPCR, quantitative PCR; RNA-seq, RNA sequencing; scRNA-seq, single-cell RNA sequencing; TEs, transposable elements.

## Interferon responses in tauopathy: microglia and beyond

A role for microglial type I IFN signaling (occurring via the IFN-α/β receptor) in AD, primary tauopathy, and associated mouse models is now relatively well established (2–5). Activation of this pathway may be regulated in part by signaling through the microglial receptor Trem2, as loss of Trem2 in a combined mutant P301L tau–PS2APP mouse model increases the likelihood of microglial polarization toward an IFN-responsive state (6). On the other hand, work involving another model of tauopathy (P301S) suggests that mutant tau-mediated expansion of IFN-responsive microglia may not depend on Trem2 signaling in all contexts (7). Additionally, induced pluripotent stem cell (iPSC) models of trisomy 21, which is a potent risk factor for early-onset AD due to triplicated *APP* (8, 9), have provided independent confirmation of a role for a microglial type I IFN response to pathologic forms of tau (10). Given that the IFN-responsive microglial state coincides with synapse loss and neurodegeneration (2, 6), and that pharmacologic and genetic inhibition of type I IFN signaling ameliorates synaptic and memory deficits in the 5XFAD model (2, 11), this subpopulation of microglia most likely contributes to pathogenesis. Interestingly, AD-related IFN responses in brain-resident cells may not be limited to microglia—brain endothelial cells in *APOE4* carriers with AD display a heightened IFN-response signature (12), and complementary findings have been observed in mouse models of amyloidosis (13). Beyond studies of mouse models and post-mortem AD brain tissue, recent integrative analyses of human genetic data and microglial transcriptomic data have suggested that variation in *OAS1*, an established IFN response gene, may modify risk for AD (14, 15).

## Interferon-responsive T cells in neurodegeneration and beyond

A unique population of highly IFN-responsive human CD4 T cells has recently been described (16, 17). A closely related population of antiviral CD4 T cells is markedly expanded in the cerebrospinal fluid (CSF) in viral encephalitis (18), and, strikingly, we have recently observed significant expansion of a very similar population of highly IFN-responsive CD4 T cells in the blood of patients with sporadic, early-onset AD (19). Excitingly, diverse physiological and pathological roles for this subpopulation continue to be suggested, as recent large-scale single-cell transcriptomic analyses have shown that IFN-responsive CD4 T cells are expanded in several autoimmune diseases including systemic lupus erythematosus (SLE) and primary Sjögren syndrome (20). Intriguingly, unpublished work also suggests that IFN-responsive CD4 T cells are expanded in patients who received hematopoietic stem cell transplants that went on to develop chronic graft versus host disease (21), suggesting these cells may have pathogenic properties. Given their relatively recent description, this population has been variably referred to as IFN-responsive T cells (16, 19), IFN signaling-associated gene (ISAG)^hi^ T cells (17, 19), antiviral CD4 T cells (18), IFN-driven inflammatory CD4 naive T cells (21), and T_naive_ *MX1* (20). This population appears to be at least partly naive (i.e., antigen inexperienced) by gene expression profiling (20, 21), raising the intriguing possibility that human IFN-responsive CD4 T cells may promote pathogenic responses in an innate-like manner, as has been shown to occur for subsets of naive CD8 T cells (22). Although it currently remains unclear if mice possess an equivalent population of highly IFN-driven CD4 T cells, several papers have found evidence for expansion of activated, IFN-responsive CD8 T cells in the brain in several AD models, including the tau P301S-*APOE4* (“TE4”) knock-in model (5) and the 5XFAD model (23). Future work in this area is required to characterize the functional role and unique properties of these IFN-responsive T cells in neurodegenerative disease.

## Interferon responses in *C9orf72* expansion carriers

The most common genetic cause of amyotrophic lateral sclerosis (ALS) and frontotemporal dementia (FTD) is a hexanucleotide repeat expansion (HRE) intronic to *C9orf72* (C9-ALS/FTD). Autosomal dominant inheritance of over 30 repeats is considered pathogenic and is associated with accumulation of repeat-containing RNA, dipeptide repeat (DPR) proteins, C9orf72 haploinsufficiency, and the development of TDP-43 neuropathology (24). Although not considered pathogenic, individuals possessing an intermediate number of hexanucleotide repeats (9–30) are thought to have an increased prevalence of systemic autoimmune conditions, including SLE and rheumatoid arthritis (RA) (25). Indeed, autoimmune diseases are more broadly enriched in patients with FTD and ALS, particularly in those with underlying TDP-43 neuropathology (26, 27). Dysregulated type I IFN signaling, in turn, has long been implicated in autoimmune conditions, including SLE, Sjögren syndrome, and a set of Mendelian disorders named “type I interferonopathies” (28). As will be described below, these findings collectively suggest that type I IFN signaling may also be dysregulated in FTD and ALS.

### C9orf72 and interferon signaling

Despite the observed relationship between immune dysregulation and *C9orf72* HREs (29), the association between *C9orf72* and IFN signaling has only recently been investigated. RNA-seq analyses of T cells from *C9orf72* knockout mice have revealed upregulation of the type I IFN response pathway, and overlapping IFN-associated pathways were also found to be increased in monocyte-derived macrophages (MDMs) derived from C9-ALS patients (30). Additionally, our group has confirmed the presence of heightened type I IFN-related gene expression in peripheral blood cells in two independent cohorts of symptomatic C9-ALS/FTD patients, compared to non-carrier controls (31). Furthermore, a 2021 study found evidence that brains donated by C9-ALS/FTD patients have increased neuronal levels of cytoplasmic double-stranded RNA (cdsRNA), and *in vitro* experiments showed that transfection of repeat-containing dsRNA led to induction of IFN-stimulated genes (ISGs). Finally, the presence of cdsRNA in mouse neurons was associated with type I IFN signaling and cell death *in vivo* (32).

### C9orf72 and STING pathway activation

Recent studies have investigated the molecular mechanisms connecting C9orf72 and IFN signaling via the stimulator of IFN genes (STING) pathway. Although a detailed description of the STING pathway is beyond the scope of this article, excellent reviews are available (33). When exposed to a STING inhibitor, both C9-ALS patient peripheral blood mononuclear cells (PBMCs) and MDMs exhibit a suppression of elevated ISG expression (30). Partial C9orf72 deficiency due to *C9orf72* HRE may thus lead to an overactive type I IFN response via upstream activation of the STING pathway. Consistent with this possibility, an independent study recently reported elevated STING protein levels in a C9orf72-deficient macrophage cell line and in C9orf72-deficient spleen (34).

### C9orf72, DNA damage, and transposable elements

C9orf72 haploinsufficiency is thought to synergize with DPR proteins in the induction of DNA damage and/or inhibition of efficient repair mechanisms (35–37). Notably, damaged nuclear DNA is an activator of the non-canonical STING pathway (38), and DNA damage is more broadly a hallmark of several neurodegenerative diseases, including C9-ALS/FTD (39). A recent study observed increased DNA damage and neuronal STING pathway activation in human post-mortem tissue and several models of *C9orf72* HRE-associated toxicity (40). Taken together, studies have identified that C9-ALS/FTD patients may possess an altered immunophenotype due to both C9orf72 haploinsufficiency and HRE-associated toxicity that is associated with heightened DNA damage and subsequent activation of the STING pathway.

Beyond frank DNA damage, IFN signaling can also be triggered by the de-repression of transposable elements (TEs), including long interspersed elements (LINEs) that cause the production of cytosolic LINE-1 cDNA (41). In symptomatic *C9orf72* HRE carriers, we observed a significant increase in the peripheral expression of human LINE-1 element *L1HS* (31). Combined with the finding that several IFN-associated signaling genes were highly upregulated in the same cohort, the dysregulation of *L1HS* in these patients suggests that TE de-repression is another potential mechanism for the enhanced innate immune response observed in *C9orf72* HRE carriers.

## Interferon responses in TDP-43 proteinopathy

Enhanced type I IFN signaling has also been observed in models of TDP-43 proteinopathy independent of *C9orf72*-related pathobiology. Physiologically, TDP-43 is involved in several facets of RNA regulation (42, 43). In disease states, TDP-43 can undergo mis-localization and post-translational modifications that promote its misfolding and abnormal aggregation in the cytoplasm (44). Depletion of TDP-43 has been implicated in regulating the accumulation of double-stranded RNA (dsRNA), promoting heightened IFN signaling (45). Additionally, recent *in vitro* experiments have shown that mislocalization of TDP-43 to mitochondria is associated with increased levels of mitochondrial reactive oxygen species (mtROS), a hallmark of mitochondrial stress (46, 47). Excessive production of mtROS in conjunction with increased mitochondrial permeability is thought to promote the errant release of mitochondrial DNA (mtDNA) into the cytoplasm (47). Cytoplasmic mtDNA, in turn, is known to precipitate the cGAS-STING cascade, initiating the activation of type I interferon signaling (46–48).

Corroborating the *in vitro* work described above, increased levels of cytosolic mtDNA have also been detected in cells derived from the spinal cord and cortex in the *TARDBP* p.A315T mouse model of TDP-43 proteinopathy. Strikingly, when the mutant TDP-43 mice were crossed with a *Sting*-deficient model, disease progression was slowed by nearly 60% and median lifespan was extended by 40% (46). Finally, direct STING inhibition increased the viability of TDP-43-mutant, iPSC-derived motor neurons; *in vivo*, Sting inhibition in the TDP-43 A315T mouse model rescued neuron loss and improved performance on a motor coordination test (46). In summary, given the observed connection between aberrant, mitochondria-localized TDP-43, mtDNA release, and STING activation, further research into STING inhibition as a potential treatment for patients affected by TDP-43 neuropathology is warranted.

## Interferon responses in Parkinson’s disease

Dysregulation of IFN signaling pathways is also implicated in the development and progression of Parkinson’s disease (PD). The underlying pathology of PD involves the accumulation of aggregated α-synuclein (α-syn) protein in vulnerable dopaminergic neurons in the substantia nigra (SN), leading to their degeneration. Recent studies suggest that α-syn dysregulation leads to activation of microglia and peripheral T cells—especially CD4 T cells—generating an inflammatory cascade, including a heightened IFN response, that may contribute to neurodegeneration in PD (49–51).

Viral infections are a well-known cause of parkinsonism, with the most famous example the postencephalitic parkinsonism associated with “encephalitis lethargica,” which was, in turn, potentially associated with the 1918 influenza pandemic (52). Certain viruses (e.g., influenza, HIV, West Nile virus) have been linked to increased risk of idiopathic PD, with *in vitro* and *in vivo* studies showing increased expression, decreased degradation, and altered post-translational modification and aggregation of α-syn, including in dopaminergic and dopaminergic-like neurons (53–56). Furthermore, α-syn has been shown to be required for effective immune response against viral pathogens. For example, both α-syn-deficient mice and human stem cell-derived dopaminergic neurons infected with neurotropic RNA viruses have higher viral loads, with increased mortality in α-syn-null mice (56, 57). Notably, using α-syn knockout human dopaminergic neurons, Monogue and colleagues showed that α-syn is required for the IFN response and effective control of viral infections, potentially through direct α-syn interaction with phosphorylated STAT2 to activate ISGs (56).

Both type I and II IFN responses have been linked to PD, although the results are mixed. Reduced type I IFN signaling mitigates neuronal death *in vitro* after treatment with rotenone, and dopamine neuron death in mice exposed to MPTP, two neurotoxins linked to PD risk (58, 59). On the other hand, mice lacking IFN-β function are reported to have increased α-syn aggregation and dopaminergic degeneration, as well as motor and cognitive deficits akin to human PD symptoms (60). Meanwhile, PD patients have elevated IFN-γ levels in the SN and striatum, as well as peripherally in plasma and CD4 T cells; IFN-γ is also secreted by subsets of PD patient CD4 T cells recognizing α-syn antigenic peptides (50, 61, 62). Overexpression of IFN-γ causes dopaminergic neuron death and nigrostriatal tract degeneration, while midbrain neuron–microglia co-cultures treated with IFN-γ neutralizing antibody show reduced microglia-mediated neuron loss when exposed to rotenone (61, 63). Altogether, these and other studies suggest that dysregulation of either type I or II IFN signaling may be a risk factor for PD.

Finally, a recent study indicates that an autoimmune response to α-syn in the gut, potentially mediated via a heightened IFN response, may directly contribute to PD symptoms (64). Circulating CD4 T cells from PD patients recognize specific α-syn epitopes, with strong affinity to α-syn_32-46_ peptide, which is highly restricted to certain human leukocyte antigen (HLA) alleles, including the PD-associated DRB1*15:01 allele. Using a mouse strain expressing DRB1*15:01, Garretti et al. showed that immunization against α-syn_32-46_ resulted in intestinal inflammation, enteric dopaminergic neuron degeneration, as well as constipation and weight loss. These changes could be partially reversed by depletion of CD4, but not CD8, T cells. Subsequent differential gene expression analysis showed upregulation of innate and adaptive immune responses, including IFN-stimulated genes (*Stat1/2, Oas2, Irf7, Isg15*). These results suggest that a dysregulated IFN response, acting in concert with CD4 T cells recognizing specific α-syn epitopes, might underlie both PD pathogenesis as well as clinical presentation.

## Interferon responses in neuropsychiatric disorders

Recent research has begun to elucidate a significant role for IFN responses in various neuropsychiatric disorders, including major depressive disorder (MDD) and post-traumatic stress disorder (PTSD). In the context of MDD, a recent single-nucleus RNA-seq (snRNA-seq) study using samples from dorsolateral prefrontal cortex has provided compelling evidence of altered IFN-γ signaling via identification of a distinct pattern of gene expression changes associated with the IFN-γ response (65). Given the higher prevalence of MDD in women and emerging evidence suggesting distinct, sex-specific molecular mechanisms in depression, this study conducted many of its analyses in a sex-stratified manner. Specifically, the authors found significant negative enrichment in IFN-γ and NF-κB signaling pathways in microglia from female cases. This supports existing literature pointing to reduced microglial activation and enhanced synaptic connectivity in females, with the reverse observed in males (66). Furthermore, alterations in microglial gene expression were more pronounced in females than males, aligning with studies demonstrating that many microglial immune functions are influenced by gonadal hormones or exhibit sex-specific characteristics (67–69). Given the inherent limitations related to the use of post-mortem tissue for snRNA-seq studies, additional research has focused on identifying peripheral blood biomarkers of MDD. For example, a large-scale, whole-blood RNA-seq study of more than 900 individuals, including cases with recurrent MDD and controls of European ancestry, revealed a significant association of MDD with increased expression of genes involved in IFN-α/β signaling, suggesting this pathway may lead to or exacerbate depressive symptoms (70).

PTSD has also been linked to aberrant immune responses, including those mediated by IFNs. A 2015 meta-analysis (71) found that elevated levels of IFN-γ, interleukin 1β, and interleukin 6 are all robust features of PTSD. Additionally, a comprehensive 2017 mega-analysis of transcriptome-wide gene expression studies involving over 500 PTSD patients and controls aimed to clarify molecular heterogeneities related to sex and trauma types (72). This study identified significant alterations in type I IFN response pathways. Notably, *IFIT3* emerged as the sole gene consistently differentially expressed across all examined trauma groups (including interpersonal-related traumas in both females and males, and combat-related traumas in males) albeit with inconsistent direction of expression change. In cases of combat-related trauma, another IFN-responsive gene, *IFI44L*, was consistently upregulated across three different studies. Furthermore, consensus gene co-expression network analyses highlighted a type I IFN signaling cascade module as enriched in all trauma groups. This underscores the importance of IFN-related molecular pathways in PTSD despite variability related to sex and trauma type in expression patterns among individual IFN-responsive genes.

## Sex differences in interferon signaling

Several studies have recorded heterogeneities in immune responses between biological males and females, including in the context of neurodegeneration and aging (73–75). Briefly, females more commonly experience enhanced immune cell activation after viral exposure and vaccination (73, 75). Many viruses exhibit sex-based differences in disease outcomes, including SARS-CoV-2 and hepatitis B, with males typically experiencing increased disease severity (76). Despite the apparent female advantage against viral exposure, females in turn experience the highest burden of several autoimmune and immune-mediated neurodegenerative diseases, including SLE (77), primary Sjögren syndrome (78), multiple sclerosis (79), and AD (80). Given that dysregulated type I IFN signaling plays a major role in autoimmune diseases (as described above), it is not surprising that sex differences also associate with differential type I IFN responses. In particular, both the differential expression of genes located on sex chromosomes as well as the production of sex hormones have been shown to alter type I IFN responses (81), but the precise immunological mechanisms underlying sex differences in the prevalence and severity of diseases associated with IFN signaling remain unclear.

Both mouse and human studies have revealed that sex may modulate IFN responses in the context of AD. In a combined tauopathy–*TREM2* R47H mouse model, only female mice demonstrated enhanced spatial memory deficits (82), and these deficits occurred amid expansion of an IFN-responsive microglia cluster. In a recent preprint, a similar mouse model was created by combining *TREM2* R47H and *APOE4* knock-in alleles in mice carrying a tau P301S transgene (83). Remarkably, female mice of this model displayed robust evidence of neurodegeneration, again in conjunction with expansion of an IFN-responsive microglial subpopulation. In a striking convergence with other recent work on tau- and TDP-43-mediated disease (46, 84), the heightened IFN response in these mice appeared to be driven by upstream cGAS–STING pathway activation. Taken together, these recent findings suggest that the effect of variation in *TREM2* and *APOE*, when combined with sex-based IFN- and other immune-mediated signaling differences, may convey unique risk for neurodegeneration in females.

In humans, using single-cell RNA-seq analysis, we discovered that female patients with sporadic early-onset AD were driving a unique peripheral immune signature (19). Specifically, female patients exhibited significantly elevated levels of a population of highly IFN-responsive T cells. We confirmed this sex difference via droplet digital PCR of isolated CD4 T cells, finding that females with early-onset AD had heightened expression of the classical IFN response gene, *MX1*. In parallel, a meta-analysis of over 1.8 million CD4 T cells found that females have increased levels of a subpopulation of naïve T cells identified by expression of the same marker gene, *MX1* (20). The exact mechanism driving sex differences in the IFN response in early-onset AD, however, remains an area of active inquiry. Interestingly, a 2022 preprint found that early-onset AD is associated with a higher prevalence of several autoimmune diseases relative to both late-onset AD and the general population (85). Whether the relative enrichment of autoimmunity in both females and early-onset AD is related to the female-driven expansion of IFN-responsive CD4 T cells in early-onset AD remains to be determined. Further investigation into the influence of sex chromosomes, hormones, and environmental factors on IFN responses in neurodegenerative and neuropsychiatric diseases is clearly warranted.

## Ancestry-associated differences in interferon signaling

In addition to sex, evidence also suggests that host genetic factors, including genetic ancestry (GA), may influence IFN signaling. GA describes the paths through which an individual inherited their DNA; in a sample, shared GA is often measured by calculating the genetic similarity between individuals to each other or to a known reference population (86). A 2021 single-cell RNA-seq study found that increasing genetic similarity to a European ancestry reference panel was associated with heightened type I IFN response after infecting PBMCs with influenza virus *in vitro* (87). Relatedly, a recent study that analyzed the post-mortem brains of self-identified Black Americans (most of whom have admixed GA) found that increasing European GA was associated with enrichment of various immune-related differentially expressed gene sets in the dentate gyrus, dorsolateral prefrontal cortex, and hippocampus. In contrast, in the caudate nucleus, increasing African GA was associated with enrichment of largely distinct immune-related gene ontology terms such as “response to virus” that are typically enriched for IFN-responsive genes. This suggests that the influence of GA on immune functions in the brain is complex and region-specific (88), and supports the notion that GA may influence IFN responsiveness *in vivo*.

Shared GA is frequently proxied by using self-reported or assumed identities that are tied to socially defined labels, such as race, ethnicity, nationality, or continental geography (89). Between these socially defined groups, multiple neurodegenerative diseases influenced by IFN signaling have disparate rates of prevalence, disease severity, and/or mortality, including AD, ALS, and multiple sclerosis (80, 90, 91). While observed health disparities may be attributed to environmental and cultural factors, including access to health care, genetic risk factors associated with shared ancestry have also been identified (92–94).

The most striking example of an IFN-associated disease with marked health disparities is SLE, which consistently displays increased prevalence and severity in non-White populations, with Black and American Indian/Alaskan Native women being the most affected groups in the United States (77). Even after controlling for socioeconomic status, studies have shown that non-White race persists as a risk factor for SLE prevalence, severity, and outcome, suggesting that there may be underlying genetic factors driving these disparities (95, 96). Furthermore, ISGs associated with SLE have higher expression levels in individuals of self-described East Asian and African American descent, in comparison to Europeans (97–99). While the exact contributions of genetic ancestry to IFN signaling in SLE remain to be elucidated, differential DNA hypomethylation of ISGs between European American and African American women with SLE has been observed. Specifically, hypomethylation of *IFI44L* was observed only in African American women (100). However, these patterns of DNA hypomethylation may also reflect risk for developing lupus nephritis (101), a severe disease outcome of SLE which European ancestry is thought to be protective against (102).

A major limitation of many studies of SLE has been the usage of race, ethnicity, or geography as a proxy for shared GA. Using socially-defined labels as proxies for shared GA relies on inaccurate assumptions about the genetic homogeneity of these groups (86). In order to more accurately quantify the contributions of population genetics on IFN signaling in SLE and beyond, it will be important to move towards continuous measures of genetic similarity, such as genetic distance (103). Furthermore, as noted in our recent systematic review, non-European ancestry populations have been significantly underrepresented in neurodegenerative disease research, particularly in genetic studies. The intentional inclusion of genetically diverse cohorts will be essential to our understanding of the host and population genetic factors contributing to IFN signaling, not only in autoimmune disease but also in the context of neurodegenerative and psychiatric disease (104).

Similar to human populations, wild-derived mice with distinct genetic backgrounds can be leveraged to explore the impact of genetic background on immune function. Indeed, a single-cell RNA-seq study of four genetically distinct, wild-derived strains of mice found that a cluster of microglia expressing IFN-responsive genes was significantly enriched in *APP*/*PS1* mice, but only on the background of the PWK strain; this effect was not observed in commonly used C57BL/6J mice or other wild-derived strains (105). Taken together, multiple lines of evidence suggest that the expression of IFN- and other immune-related genes is potently influenced by genetic variation.

## Interferon responses in aging

Beyond biological sex and ancestry, the process of aging is thought to exert a major effect on the IFN response. This phenomenon, explored recently in several excellent review articles (106, 107), has been studied primarily in model systems (108) but is also likely to play an important contributory role in human diseases of aging such as AD. While some studies have found elevated type I IFN responses associated with aging (e.g., at the choroid plexus (108)), others have found decreased IFN signaling potential (e.g., in peripheral myeloid cells (109)). Given that inappropriate IFN pathway hyperactivation may underlie diseases of premature aging (110), it is reasonable to hypothesize that aging-associated neurodegenerative diseases are also driven, in part, by augmented IFN signaling. How these findings can be reconciled with the known, aging-associated reduction in peripheral innate immune IFN signaling (109, 111) remains an important open question worthy of careful future investigation.

## Clinical and neuroimaging features of interferon response

While there has been marked progress in our understanding of how dysregulated IFN signaling contributes to neurologic disease in model systems, there is relatively limited information on its contribution to behavioral symptoms and risk for neurodegenerative disease in human populations. In this section, we provide a focused summary of the clinical and neuroimaging features of IFN response as they relate to neuropsychiatric and neurodegenerative disease.

### Clinical features of interferon treatment and response

Our clinical understanding of the behavioral symptoms of IFN-pathway dysregulation stems from two key clinical populations: patients receiving exogenous IFN therapeutically and patients with intrinsic interferonopathies. Interestingly, some of the earliest clinical evidence that IFN dysregulation caused neuropsychiatric symptoms was incidentally discovered during IFN treatment for hepatitis C, hepatitis B, and melanoma (112–114).

Several key features of IFN-related neurotoxicity are shared – depression and fatigue are the most common behavioral symptoms and occur in up to 70% of patients while rarer symptoms such as mania, seizures, and psychosis occur in less than 5% (112, 115). These symptoms have been most thoroughly characterized in the setting of IFN treatment (112), but overlap with behavioral symptoms in multiple diseases including primary immune-mediated diseases such as HIV-associated neurocognitive disorder (HAND) (116, 117) and subsets of SLE (118), neuropsychiatric diseases such as major depression (119) and schizophrenia (120), and neurodegenerative diseases like AD (80). In the case of HAND, type I IFN responses are thought to be important for maintaining virological control, both systemically and in the brain (121, 122), but may also contribute to cognitive dysfunction. Multiple mechanisms have been hypothesized to explain the connections between IFN pathway dysregulation and psychiatric symptoms including increased degradation of serotonin, decreased synthesis of dopamine, and altered glutamatergic signaling (123), all of which are key regulators of mood and behavior. Interestingly, an additional modulator of clinical symptoms has been hypothesized in neurodegenerative diseases – IFN indirectly promotes NMDA receptor activation, a possible driver of neurotoxicity and worsening symptoms (123).

Treatment of the behavioral effects of interferonopathies remains in its infancy with many promising treatment paradigms though few validated treatments. For example, tyrosine kinase inhibitors have successfully treated Aicardi–Goutières syndrome (AGS) (124–126), an inherited type I interferonopathy which presents with immune dysfunction, motor deficits, and developmental delay (127). Whether the findings from inherited syndromes can be extrapolated to IFN-related neuropsychiatric and neurodegenerative diseases is an exciting area for future research.

### Neuroimaging features of interferon response

Neuroimaging is an important though underutilized tool in the study of interferonopathies, highlighting promising links between the above-described clinical symptoms and the underlying IFN-mediated pathobiology described elsewhere in this review.

Exogenous IFN administration has been shown to alter brain structure and function in both patient populations and healthy controls. Patients undergoing IFN-α treatment demonstrate decreased functional activity in basal ganglia during reward processing tasks (128), increased amygdala activity during emotion processing tasks (129), and impaired whole-brain connectivity during resting state MRI (130). Furthermore, early imaging evidence indicates that dopamine and glutamate concentrations are altered in basal ganglia and that these changes correlate with functional connectivity and depressive symptoms (128, 131). Remarkably, IFN-β administration in healthy controls also decreases functional activity in the ventral striatum during reward processing tasks and amygdala during emotion-recognition tasks (132). Interestingly, one quantitative MRI study in healthy controls demonstrated that IFN-α administration alters the intrinsic T1-relaxation values (a measure of tissue composition impacted by factors such as protein levels, water content, etc) for hippocampus and that these changes correlated with markers of decreased blood brain barrier integrity (133). Despite the methodological differences, these findings suggest that the effects of administered type I IFN are likely independent of disease status, point to neuroanatomic pathways by which IFN alters behavior, and further highlight the importance of dopamine in the brain’s response to IFN.

Compared to imaging studies of exogenous IFN administration, there are relatively few studies of intrinsic interferonopathies. While uncommon, inherited interferonopathies such as monogenic SLE and AGS most commonly present with intracerebral calcifications and T2 white matter hyperintensities (134, 135). Not surprisingly, imaging findings are variable depending on each syndrome’s underlying genetic mutation (134). For example, AGS patients with *SAMHD1* mutations may present with intracranial vasculopathy while patients with *ADAR1* mutations present with striatal necrosis (134). There is limited literature directly testing whether IFN levels alter brain structure or function in neurodegenerative disease, but indirect evidence suggests that IFN signaling plays an important role in brain structure in C9-FTD (31). Future studies will be required to determine whether IFN levels are associated directly with neuroanatomic correlates of neurodegenerative disease progression.

## Overlapping interferon pathway dysregulation in neurodegenerative and psychiatric disease: the way forward

In summary, we have reviewed key evidence from both basic and translational scientific approaches to show that aberrantly upregulated IFN and downstream IFN signaling not only predispose patients to neurodegenerative diseases but may also modulate risk for psychiatric diseases. Indeed, the links between IFN and both neurodegenerative and neuropsychiatric diseases go far beyond overlapping clinical features and behavioral symptoms. At the molecular level, broad-ranging data from mice, human brain tissue, and even neuroimaging studies suggests that IFN-mediated neurotransmitter dysregulation, particularly dopamine and glutamate dysfunction, is a shared risk factor for psychiatric symptoms in both neurodegenerative and neuropsychiatric diseases (56, 60, 128, 131). Future studies should aim to directly address the question of whether IFN pathway dysregulation in the context of neurodegeneration drives psychiatric symptoms in addition to cognitive decline.

At the cellular level, converging evidence from mouse and human studies suggests that microglia play a key role in IFN pathway dysregulation and that microglial dysfunction associates broadly with risk for neurodegenerative diseases like AD (4, 82, 83) and PD (61) alongside sex-specific effects in psychiatric diseases like MDD (66). Beyond the large body of literature connecting aberrant microglial IFN signaling in neurodegenerative disease, additional evidence points to important roles for endothelial cells (12), the choroid plexus (136), and IFN-responsive CD4 and CD8 T cells (5, 19, 23) in neurodegenerative disease. IFN-responsive CD4 T cells remain poorly understood and are particularly intriguing given their expansion in sporadic early-onset AD (19), viral encephalitis (18), and autoimmune disease (20). That autoimmune disease is found at higher-than-expected prevalence in early-onset AD (85) suggests a mechanistic explanation for dysregulated peripheral IFN signaling in early-onset AD and the potential for shared immune-mediated etiology, although additional study is needed to confirm this possibility.

The primary source(s) of type I IFN for modulating T cell and microglial activity in the context of neurodegenerative and psychiatric disease represents an important yet unresolved question. Plasmacytoid dendritic cells (pDCs) are a major source of type I IFN in the context of viral infection (137), but their role in producing IFN during neurodegeneration remains unclear. Intriguing recent work suggests that a pathogenic variant in *TLR9* may represent a novel, autosomal-dominant cause of early-onset AD (138). Interestingly, TLR9, while robustly expressed in pDCs, is apparently not expressed in human microglia (138). Strikingly, TLR9 is a major regulator of type I IFN production by pDCs (139). Taken together, these findings suggest that dysregulated production of type I IFN by pDCs may promote risk for early-onset AD. Further research into the potential role of TLR9 signaling in AD, including the identification of additional families with early-onset dementia harboring pathogenic *TLR9* mutations, is now needed.

Beyond these connections, many psychiatric symptoms in primary autoimmune diseases such as SLE and Sjögren syndrome are also increasingly attributed to microglial dysfunction (140, 141). At the network level, early evidence has shown broad, IFN-associated dysregulation of genetic networks (3), linked inflammatory cytokines (120), and even functional neuroanatomic networks (130). Most relevant network analyses to date, however, have focused relatively narrowly on a single data modality or disease state. It stands to reason that combined analyses of multiple data modalities (e.g., genetic and functional brain networks) and disease states (e.g., neurodegenerative, primary autoimmune, and psychiatric) will reveal scientifically and clinically relevant information about the shared biology underlying these complex diseases. With large and publicly available multimodal datasets available for these diseases, integrative analyses represent a promising, even vital, area for future research.

There is now well-established evidence for type I IFN pathway dysregulation—generally manifesting as pathologic upregulation of IFN-response genes—in a range of neurodegenerative and neuropsychiatric diseases. In addition, clear evidence shows that recombinant IFN-α/β treatment can promote cognitive and behavioral dysfunction. Given the reasonable hypothesis that aberrant IFN signaling may exacerbate pathology and contribute to cognitive dysfunction and/or decline in these diseases, is it similarly reasonable to postulate that inhibiting excessive IFN signaling would represent an attractive therapeutic strategy? A monoclonal antibody that inhibits signaling through the IFN-α/β receptor has been approved by the United States Food and Drug Administration (FDA) for use in SLE and could represent a promising candidate for early clinical testing in neurodegenerative disease. Caution is warranted, however, given the reported increase in herpes zoster (142, 143) and influenza infections (144, 145) in those being treated with this antibody. Because viral infections may also increase risk for neurodegenerative disease (146), this therapeutic strategy could perversely promote incipient neurodegeneration and cognitive decline. Indeed, a precise homeostatic balance in IFN signaling may be required to enable effective antiviral responses throughout life while, at the same time, not unnecessarily promoting risk for neurodegeneration.
